# The Diagnosis of Primary Ciliary Dyskinesia: Putting The European Respiratory/American Thoracic Guideline Into Practice

**DOI:** 10.1002/ppul.71577

**Published:** 2026-03-26

**Authors:** Katharine Harman, Amjad Horani, Amelia Shoemark

**Affiliations:** ^1^ Department of Paediatrics Kings College Hospital NHS Foundation Trust London UK; ^2^ PCD Diagnostic Centre Royal Brompton Hospital London UK; ^3^ Department of Cell Biology and Physiology Washington University School of Medicine Saint Louis Missouri USA; ^4^ Department of Respiratory and Gastroenterology University of Dundee Dundee UK

## Abstract

Primary ciliary dyskinesia (PCD) is a rare inherited disease which is characterised by progressive lung disease, chronic rhinosinusitis, repeated middle ear infections, laterality defects, and reduced fertility. An early diagnosis is critical to reduce morbidity, however diagnosis is often delayed due to the heterogeneity in clinical presentation. Diagnosis relies on multiple tests, and the American Thoracic (ATS) and European Respiratory (ERS) societies have previously developed separate diagnostic guidelines. Both differed in recommendations and approach. The recently published joint ERS/ATS guidelines for the diagnosis of PCD is the first evidence‐based guidelines that unifies the approach recommendations between both societies. These guidelines were formulated by a task force (TF) comprised of experts in the field, and were guided by a systematic reviews and GRADE (Grading of Recommendations, Assessment, Development and Evaluation) approach. The TF formulated three 'Patients, Intervention, Comparison, Outcomes' (PICO) questions to determine the accuracies of (1) nasal nitric oxide (nNO) (2) high‐speed video microscopy (HSVM) and 3) immunofluorescence (IF) when compared to a reference test of either transmission electron microscopy (TEM) and/or genetics. There were also three narrative questions which sought to determine (1) what clinical presentation would support a clinician to refer a patient for PCD diagnostic testing (2) what additional diagnostic tests could be useful and (3) how to overcome PCD diagnostic challenges in resource limited settings. This review presents example cases that highlight the recommendations of the clinical practice guidelines.

## 1 Introduction

### 1.1 What is PCD?

Primary ciliary dyskinesia (PCD) is an inherited disease characterised by dysfunctional motile cilia. Motile cilia are organelles located on the apical epithelial surface of the upper and lower airways, as well as other organs including the reproductive organs, and the embryonic node which is responsible for determining laterality. There are approximately 200 motile cilia on the surface of airway multiciliated epithelial cells. Their primary function is to propel liquid and mucus. The structural and functional ciliary abnormalities in PCD result in disruption of the coordinated movement of the cilia. In the airways this results in impaired mucociliary clearance (MCC) and an accumulation of mucus which leads to bacterial colonisation, chronic inflammation, and recurrent sino‐oto‐pulmonary infections characteristic of PCD [1]. Most PCD patients develop early‐onset bronchiectasis and progressive lung disease which can lead to respiratory failure [2].

Dysfunction of the embryonic nodal cilia can cause laterality defects including situs inversus totalis, dextocardia, and heterotaxy syndromes. The latter is often associated with complex congenital heart disease. Male Infertility is also common due to defects in the sperm flagella as well as motile cilia that line the efferent ducts. Females are often sub‐fertile due to ciliary dysfunction in the Fallopian tube [3]. There is a rare possibility of hydrocephalus/ventriculomegaly in PCD due to dysfunction of ependymal cilia in the CNS [2].

### 1.2 PCD Is Often Underdiagnosed/Diagnosed Late

PCD is a rare condition with an estimated prevalence of 1:7500 [4]. Disease prevalence can vary in different regions, and maybe higher in communities with high rates of consanguinity [4]. The figures from the European research network registry (ERN‐LUNG) and North American registry however suggest that many people with PCD remain undiagnosed [5].

Late or missed diagnosis can be due to lack of awareness or disease recognition. Patients with PCD have clinical symptoms which are common and non‐specific particularly in paediatrics. Symptoms also frequently overlap with other conditions, such as cystic fibrosis (CF), immunodeficiencies and asthma. As a result, patients frequently visit physicians' multiple times prior to being referred for specific PCD diagnostic studies [6].

### 1.3 Tests Used to Diagnose PCD

There are no gold standard tests for PCD diagnosis. Accurate diagnosis requires the use of a combination of multiple tools which are performed at highly specialist centres. Diagnostic practices vary depending on available expertise and resources. The 2 commonly used confirmatory tests are genetic testing and axonemal ultrastructure analysis using transmission electron microscopy (TEM), both of which feature in the ERS and ATS guidelines [7].

### 1.4 Genetics

As PCD is a genetic condition, most experts agree that identifying pathogenic genetic variants in a known gene associated with PCD is sufficient to confirm a diagnosis. PCD is most commonly inherited in an autosomal recessive pattern, although X‐linked and dominant forms unique to specific genes have been reported. To date there are over 65 genes that have been associated with PCD. It is important to emphasize however, that there are varying degrees of evidence for disease causality. To cope with the number of reported genes associated with PCD, a group of experts have been evaluating the evidence to curate these genes. A list of curated confirmed and disputed genes can be found at https://www.clinicalgenome.org/affiliation/40102/ or https://beat-pcd.squarespace.com/research.

It is estimated that 70% of patients with PCD have an identified biallelic variant in a known gene associated with PCD. As more accurate genetic tools become available new genes will be identified. When a genetic confirmation is not achieved, additional tests are required to support a diagnosis.

### 1.5 TEM

TEM has historically been the ‘gold standard’ tests for PCD and in the 2017 ERS guideline was demonstrated to be 99% specific in confirming a PCD diagnosis if performed in a specialist centre [8]. However, TEM analysis is time consuming, requires significant expertise and can be normal in 20%–30% of people with genetically confirmed PCD. TEM has a place in supporting a diagnosis of PCD when genetic results are inconclusive or not available as occurs in approximately 30% cases TEM is not a substitution for genetics and genetic confirmation should be achieved whenever possible.

TEM results can be categorised into 2 classes of defects, based on the observed ultrastructure changes [9]:

- Class 1 defects which can confirm a diagnosis of PCD, include:
  ◦ Outer dynein arm, inner and outer dynein arm, and inner dynein arm and microtubular disorganisation.
- Class 2 defects which are suggestive of PCD and require supporting evidence from other diagnostic modalities, include:
  ◦ Outer dynein defects in less than 50% of cilia cross‐section studies, central pair defects, microtubule disorganisation with inner dynein arms present and reduction in ciliation with micro localisation of basal bodies.

Additional investigations are frequently performed in order to confirm or exclude a diagnosis of PCD including nasal nitric oxide (nNO), high speed video microscopy (HSVM) and immunofluorescence staining (IF).

### 1.6 Is Confirming a PCD Diagnosis Important?

Early diagnosis of PCD improves outcomes (10). Earlier diagnosis (age < 8) is associated with improved lung function throughout childhood [10]. Confirming the diagnosis of PCD allows patients to be managed by specialised multidisciplinary teams with treatment of infections, airway clearance, and expert management of complications.

Furthermore, although currently there are no PCD‐specific approved therapies that restore ciliary function, there are ongoing pre‐clinical studies with mRNA correction of genes associated with PCD. Other approaches such as gene editing and readthrough therapies are also under development. Therefore, a genetic diagnosis is of utmost important to identify which patients are eligible for trials for possible curative therapies.

### 1.7 Why Unified New Guidelines Were Developed?

Until the publication of this new joint ERS/ATS guidelines, there were separate published guidelines [8, 11]. Both were evidence‐based but were out of date due to advances in cilia genetics and the development of novel diagnostic techniques. Moreover, there was a need for a unified international guideline to standardise diagnostic approaches and hence care delivery. A unified approach also allows for better integrated data collection through registries, which is essential to define the natural history, define rare symptoms, and characterise morbidity especially in adult populations with PCD. A unified approach to diagnosis is of great importance for designing collaborative international research and trials which is essential in rare diseases.

This article highlights key points of the new 2025 diagnostic guideline and includes illustrative case studies.

### 1.8 Who to Refer for Testing?

One of the main difficulties for clinicians is identifying which patients should be referred for PCD diagnostic testing. As described, symptoms associated with PCD overlap with more common conditions and are not unique to PCD. Here, it should be emphasised that it is not a specific symptom that should trigger evaluation for PCD but rather a combination of symptoms or the patterns of clinical presentation. To assist clinicians in this decision the ERS/ATS task force considered what are the clinical manifestations in the newborn period, childhood, and adulthood that are often seen in PCD patients. The main features are highlighted in Box 1.

BOX 1 **Relevant features in the clinical history**

- Neonatal respiratory distress at term.
- Admission to neonatal care unit for persistent supplemental oxygen need.
- Situs anomalies.
- Persistent wet cough.
- Persistent perennial rhinitis.
- Chronic ear or hearing problems.
- Early onset bilateral bronchiectasis of the middle/lower lobes.
- Male and female infertility.

Useful tools to prioritise to patients for referral for PCD diagnostic studies based on clinical symptoms

- PICADAR for use in patients with a persistent wet cough [12].
- ATS clinical criteria [13].
- Clinical criteria from the ERS adult bronchiectasis guidelines [14].

Furthermore, there are two symptom‐based tools that can support non‐experts to identify individuals likely to have PCD:

1. The PrImary CiliARy DyskinesiA Rule (PICADAR) is a seven‐point questionnaire which has been validated in paediatric and adult cohorts to predict who will be most likely to have a positive diagnosis of PCD [12]. Importantly, all individuals must have a baseline a persistent early onset wet cough for the tool to work accurately. The score is based on neonatal chest symptoms at term, admission to NICU, situs anomalies, congenital heart defect, persistent perennial rhinitis and chronic ear or hearing problems.

1. The ATS clinical criteria developed by *Leigh* et al provides an alternative tool which combines unexplained neonatal distress, early onset year‐round cough, early onset year round nasal congestion and laterality defects to distinguish which patients may have a diagnosis of PCD [13].

The first case described has clinical symptoms associated with PCD and illustrates how use of a scoring algorithm can help in prioritising who to refer for PCD investigations. It is important to note these tools are imperfect, especially in adult cohorts and in those with novel genotypes where validation is required.

### 1.9 Case 1

A 4 year old girl was referred for recurrent wet cough. She was born at term. Prenatal history was not significant, but she developed increased supplemental oxygen requirement at 24 h of age, and needed care in the neonatal care unit for about 10 days. No clear etiology for supplemental oxygen need was identified.

Parents report she had always had nasal congestion and a wet cough which was present from a few months of age. She received numerous courses of oral antibiotics for bronchitis with temporary improvement. Parents report that she had numerous episodes of otits media that required oral antibiotics. They have concerns that she has hearing loss as speech development is delayed.

She attends childcare 3 days a week and is fully immunised. Her parents were not related to each other and there is no significant family history including for cystic fibrosis, immunodeficiency, or cardiac problems.

Physical examination revealed a well grown child. She has a spontaneous wet cough. No chest wall deformity, digital clubbing, or respiratory distress were observed. A chest radiograph was interpreted as normal non‐specific broncho‐vascular markings likely related to a viral infection.

She was referred for further evaluation due to symptoms of chronic cough.

Using a symptoms based score, this child had a score of 8 on PICADAR, and 3 out of 4 on the cardinal features described by Leigh. Based on these scores, further testing for PCD was performed.

The patient was unable to perform nNO tests. Genetic testing revealed bi‐allelic pathogenic variants in *DNAH5* in trans. TEM analysis revealed absent outer dynein arms (ODAs) in the ciliary axonemes. She was referred for management of PCD at her local specialist centre.

This case illustrates the use of a clinical scoring system to prioritise this child for PCD diagnostic testing. Although both symptom‐based tools are more likely to identify individuals with laterality defects, they help prioritise other patients with non‐specific symptoms. This is vital as there are an increasing number of genes identified which are not associated with situs abnormalities.

The guideline also states that motile cilia dysfunction is being increasingly diagnosed in symptomatic patients who have variants in genes previously known to cause syndromic ciliopathies with additional clinical characteristics such as retinitis pigmentosa and skeletal abnormalities. Clinicians should be aware that patients may present with atypical and a broad range of symptoms.

### 1.10 What Tests Should Be Performed?

After identifying that a patient has a high risk of having PCD, it is recommended to refer them to a PCD specialised diagnostic centre for further evaluation. A number of different investigations can be performed in addition to Genetics +/− TEM Recognising the strengths and limitations of each investigation is essential when interpreting the results. Patients can be classified as either (1) confirmed PCD, (2) PCD highly likely (3) suspected PCD or (4) PCD highly unlikely. The three most common additional tests are nNO measurement, high‐speed video microscopy (HSVM), and immunofluorescent analysis (IF). The availability of these tests vary by centre with some having access to all or limited additional investigations.

Evidence show that nNO, HSVM and IF staining are all valuable and can be included in a PCD diagnostic testing algorithm, in no particular order, in addition to genetics and/or TEM. The guidelines emphasise that:

No single test has 100% specificity and sensitivity which necessitates the use of multiple tests in a diagnostic approach.

There is no evidence for using any single test in any order, however practical considerations may mean one is done before others.

### 1.11 These Tests Are Considered in Turn

#### 1.11.1 Nasal NO (nNO)

The measurement of nNO is non‐invasive and quick. Low nNO levels are described in patients with PCD. Measurement requires use of a chemiluminescent measurement device to detect nitric oxide levels through one or both nostrils, following standardised techniques. There are two methods to measure nNO: during velum closure (breath hold or exhalation against resistance) or during tidal breathing which is used in patients younger than 5 who are unable to perform velum closure breathing [15]. The guidelines consider both procedures as viable, but emphasised that measurement during tidal breathing is less accurate and more variable [7]. A new study published since the guideline suggest nNO cut off values that can be used in infants and young children [16].

The guidelines recommend the use of nNO to support a diagnosis of PCD in concordance with other tests. The studies that were reviewed were performed in patients who were referred for PCD diagnostic testing with a good clinical history and therefore high pretest probability. Therefore, nNO should not be used as a screening tool in the general population, as the false positive results would be high [17].

For the measurement of nNO using velum closure technique the recommendation was strong with moderate certainty of evidence based on 5 studies and with high sensitivity and specificity using the standard cut‐off value of 77 nL/min. Only 3 studies assessed the measurement of NO using tidal breathing. Analysis of these studies revealed reduced sensitivity and specificity compared to the velum closure manoeuvre. The recommendation is therefore conditional with very low certainty of evidence for use of nNO measurement using tidal breathing, and this technique risks a larger number of non‐PCD patients being misclassified as PCD. It is suggested that the velum closure technique be used whenever possible, with tidal breathing reserved for cases when measurement using velum closure is not possible.

### 1.12 High Speed Video Microscopy Analysis (HSVM)

High‐speed video analysis uses light microscopy to analyse the cilia beat pattern and frequency of airway multiciliated epithelial cells. This can be performed immediately after taking nasal or bronchial brush biopsies or after regeneration of ciliated airway epithelia in cell culture, which is performed at highly specialised centres [18, 19]. The guidelines reviewed both techniques separately. HSVM is unique in that it's the only diagnostic test in which ciliary dyskinesia can be directly visualised. Specific features including reviewing the beat pattern (effective forward and recovery ciliary beat or areas of statis or rotation), amplitude, mucociliary clearance, and frequency.

The addition of HSVM to the diagnostic guidelines was strongly recommended with very low certainty of evidence and advised to be used in combination with other tests and not as a stand‐alone investigation. This was based on 8 studies in total which revealed high sensitivity and specificity of the test. Post culture assessment is more sensitive and specific than analysis of freshly obtained samples. However, these tests can be costly ranging from several hundred dollars up to several thousand depending on whether invitro culture is performed. Moreover, HSVM requires specific expertise and equipment that may not be available in all centres.

HSVM can be useful especially when other tests are inconclusive. Its utility is seen in cases when TEM is inconclusive for example as seen with variants in *DNAH11, HYDIN, RSPH1* [20, 21, 22]. Prior to discovery of these genes many patients with a highly likely diagnosis were followed up due to high‐speed video patterns, and there remain further cases with persistent HSVM changes after culture awaiting further improvements in genetic diagnosis capabilities.

### 1.13 Immunofluorescence (IF)

IF is performed on the nasal brushing samples of patients. A panel of fluorescently tagged antibodies are applied to identify absent or mis‐localised proteins correlating with patterns caused by PCD‐associated genes [23]. The guidelines strongly recommended the use of IF with a high certainty of evidence.

This recommendation was based on 2 studies which showed that IF provided timely and accurate diagnostic results for many patients evaluated for PCD and did not add to test burden [24, 25]. Sensitivity and specificity were very high in both studies. The use of IF can be particularly useful in resolving cases where genetic results are inconclusive as occurs with variants of unknown significance (VUS), particularly for genes which are known to be associated with normal or near normal TEM.

### 1.14 Case 2

A 6 year old boy was referred for evaluation due to recurrent wet cough. He was born preterm at 35 weeks of gestation, and parents reported recurrent lung and ear infections since he was 18 months old – soon after he started attending day care. Parents report that the cough is frequent but does not occur every day.

Parents are non‐consanguineous. There is history of an uncle who had died as a child due to a cardiac disease.

Physical examination revealed he was small for his age with weight and height both below the 3rd percentile. He had a spontaneous wet cough. Early signs of digital clubbing were observed. Lung exam revealed scattered rhonchi. A chest radiograph was interpreted as normal, however a prior chest radiograph revealed bilateral patchy infiltrates.

Due to concerns that symptoms may be related to PCD, the patient underwent nasal NO measurement using a velum closure technique which was 62 nL/min. PCD genetics using a panel of genes was negative. TEM analysis of a nasal brush was interpreted as normal. IF analysis of a nasal sample using a panel of antibodies did not identify abnormalities. High‐speed video microscopy using cultured airway cells showed a normal beat pattern in the majority of the sample.

Based on the above tests, a diagnosis of PCD unlikely was made, and further evaluation was performed. Subsequently a diagnosis of primary immunodeficiency was made.

This case highlights the value of multiple tests. Although the low nNO initially suggested he might have PCD, all other tests were negative. Moreover although these symptoms may occur in PCD they started relatively late. All together, these findings allowed the clinicians to consider him to be ‘highly unlikely PCD’.

Nasal NO has also been reported to be reduced in other conditions such as immune deficiencies and cystic fibrosis [26], and these conditions should be considered during the evaluation of PCD patients especially when symptoms are highly suggestive, as they can lead to specific and lifesaving therapies.

### 1.15 Case 3

A 13 year old boy who was first referred at the age of 5 years for recurrent wet cough, is being evaluated for persistent nasal congestion, chronic otorrhea, and recurrent otitis media. His growth and development were normal. He has an elder sister with similar symptoms.

Patient was born at term and required supplemental oxygen for 5 days after birth.

Physical examination revealed crackles on auscultation of the right lower lobe. No digital clubbing was appreciated. A chest radiograph revealed non‐specific bronchial wall thickening.

Testing for PCD included measurement of nNO with a velum closure procedure, which was 20 nL/min. TEM analysis did not reveal ultrastructure abnormalities. High‐speed video showed disorganised beat pattern with cilia showing a rotational movement with limited clearance. Genetics analysis revealed two variants of unknown significance in *HYDIN* gene, inherited in trans from both parents. IF showed absent localisation of *SPEF2*.

Based on the clinical symptoms, positive adjunct test, but lack of definite genetic testing, he is designated as PCD highly‐likely.

Re‐evaluation of the genetic evidence allowed the VUS to be upgraded to pathogenic based on the additional available in vitro tests. This allowed the designation of this patient to be changed to *confirmed PCD*.

Case 3 demonstrates the utility of multiple tests when investigations are inconclusive. IF is helpful in cases where genes are associated with normal or near‐normal electron microscopy (other examples include *DNAH11*). Confirming the specific genetic cause of PCD will be relevant when genetic therapies become available.

When a diagnosis of PCD cannot be confirmed using genetics or TEM, additional tests are needed. Not all tests are always needed, however limiting the number of tests may reduce the diagnostic accuracy. The guideline strongly advises that the quality and technique of the test conducted follow accepted standards. Referral to an expert centre for diagnosis should be conducted whenever possible.

### 1.16 How Do We Facilitate Diagnosis PCD in Resource Limited Areas?

The guideline described a multiple‐test approach to diagnosing PCD which requires highly specialised teams and the availability of investigations which are not available in resource limited settings.

Six studies addressed diagnostic strategies in resource limited settings. Two main areas were identified to aid PCD diagnosis in these settings. The first focused on utilising the previously described symptom tools such as PICADAR or the scoring tool by Leigh et al. [12, 13]. These are cheap and evidence‐based to help prioritise which patients have a high likelihood of having PCD necessitating further tests. This recommendation was based on a study in Egypt, however further validation in other settings outside the UK are needed [27]. It should be emphasized that the scoring tools are only screening tools and should not be used to rule out PCD. These tools will likely be updated to better capture PCD cases in light of newly described genes associated with PCD.

The other emphasis is on the importance of international collaboration for PCD diagnosis. In centres/countries with inadequate funding for genetic testing and insufficient healthcare system infrastructure it was shown that collaboration with established centres help with education, procedural training, diagnostic expertise, and access to funding mechanisms for expanded genetic testing. Networks such as the BEAT‐PCD, ERN‐LUNG, the Genetic Disorders of Mucociliary Clearance Consortium, and patient foundations can help with the diagnosis and management of PCD by supporting local physicians.

### 1.17 Future Methods for PCD Diagnostics

Although the best evidence was used at the time of writing of these guidelines, there remain knowledge gaps in the diagnosis of PCD.There are a number of improvements and alternative methods being trialled in research environments which may improve PCD diagnosis in the future. These include the use of radiolabelled mucociliary clearance, optical coherence tomography, improvements in microscopy technology for TEM and IF, digital augmentation of images by machine learning techniques, new cell culture techniques, and advances in genetic data acquisition and analysis [28, 29, 30, 31, 32].

## 2 Conclusion's and Future Perspectives

The recently published ERS/ATS joint guidelines for the diagnosis of PCD aim to assists health professionals in the pathway of diagnosis in a patient with PCD from the initial clinical presentation to which tests to perform. The guideline also covers how this can be extrapolated to resource poor areas. By achieving early and accurate diagnosis we can optimise mangement in children, preserve lung growth, and minimise and prevent complications.

## Author Contributions

Writing and approval of manuscript: all authors.

## Funding

The authors received no specific funding for this work.

## Conflicts of Interest

Amelia Shoemark reports grants from AstraZeneca and Lifearc; consultancy fees from Spirovant, Translate Bio and ReCode Therapeutics; payment or honoraria for lectures, presentations, manuscript writing or educational events from Translate Bio, Ethris and Insmed; a leadership role as Chair BEAT‐PCD ERS CRC; and involvement in European Respiratory Society Clinical Research Collaborations (EMBARC and AMR Lung). Amjad Horani reports leadership roles with the PCD Foundation and PCD Research. Katharine Harman has no potential conflicts of interest to disclose.

## Educational Aims

This article is intended for those involved in assessing and managing patients with wet cough/bronchiectasis and would refer patients for PCD diagnostic testing. It aims to inform:

- Who to refer for PCD diagnostic testing.
- What tests are available and how they can be used clinically to diagnose or exclude PCD.
- How to optimise diagnostics in resource limited settings.

## Data Availability

Data sharing not applicable to this article as no datasets were generated or analysed during the current study.
